# A Manual of Animal Vaccination

**Published:** 1889-03

**Authors:** 


### Reviews of Books.

---

*A Manual of Animal Vaccination.* By Dr. E. WARLOMONT. Translated and edited by ARTHUR J. HARRIES, M.D. London: J. & A. CHURCHILL. 1885.

*Vaccination Vindicated, being an Answer to the Leading Anti-Vaccinators.* By JOHN C. McVAIL, M. D., D.P.H. Cambridge. London: CASSELL & COMPANY, Limited. 1887.

*A Pamphlet on Vaccination.* By WM. WOODWARD, M.D. London: SIMPKIN & MARSHALL. 1888.

WE owe an apology to the author and translator of the first named of these works for our tardy recognition of a book which, in the present disturbed state of the public mind, should be in the hands of every medical practitioner, whether he is likely to be called upon to perform the operation of vaccination or not.

Nearly the first half of the work is taken up with "General Observations on Vaccination," in which its history and the questions relating to humanised lymph are conveniently summarised. But it is the second part—"concerning Animal Vaccination"—which gives the book its special interest. The needless fears about the transmission of syphilis are of course banished by this method; and, on the question of inoculating tubercle by the vaccination-process, Dr. Warlomont insists upon the impossibility of doing "so by means of *superficial* insertion of its bacilli." The author sums up the matter thus "Vaccination is free from danger when it is practised with necessary precautions, skill and care. It is not more harmful than piercing the ears to place rings in them."

We regret to say that the book is without an index, thus, inviting unfavourable comparison with Dr. Seaton's classic work on Vaccination.

Dr. McVail's book is of a kind different from the preceding, but of eminent value. It is a monument of industry, consisting of statistics of the most useful description, in reference to the power of vaccination in mitigating smallpox. It is a work indispensable to anyone engaged in the vaccination controversy, and is furnished with an admirable index, much facilitating the use of the copious figures by which the arguments of the book are substantiated. In a certain sense, it is unfortunate that in proportion to its good results the importance of vaccination fails to be recognised, as the more remote we get from smallpox epidemics the less we are able to realise our indebtedness to the prevailing efficacy of this operation. Dr. McVail has done the community a service, in preserving, and presenting in a convenient and accessible form, the overwhelming evidence of the benefits of vaccination.

Dr. Woodward's pamphlet consists of a paper read by him before the Worcester Young Men's Christian Association. It is very useful in its way, and is to be commended as an active Public Vaccinator's earnest defence of the value of vaccination.

Vaccination has got into undeserved discredit by the way in which its details have been carried out by thoughtless or careless operators. It is much to be desired that all vaccinations should be taken out of the hands of private practitioners, and allowed to be performed only by public vaccinators. The difficulties in the way of this much-needed reform could be easily overcome. Vaccination, as an important branch of preventive medicine, should be under Government inspection. Not only is there great difficulty, privately, in obtaining trustworthy lymph, often necessitating a resort to unauthorised sources, but, in deference to the sentimental objections of ill-informed parents there are many practitioners of good social standing who are not ashamed, by vaccinating by one or two small insertions, to earn a cheap popularity, although thereby a serious danger is added to the life of a child thus made unfit to successfully resist a possible attack of smallpox. There are also doctors of a lower grade who set themselves up in unwholesome opposition to the Public Vaccinator, and, by performing the operation for a degrading fee of sixpence or a shilling, with a vaccination also much reduced in quantity—and therefore in quality—draw off a considerable number of ignorant mothers from the Vaccination Station, the efficiency of which becomes impaired through a greatly diminished attendance seriously limiting the selection of lymph, and the proper management of which becomes well-nigh impossible.

In the light of Marson's figures (Seaton's *Handbook of Vaccination*, ed. 1868, p. 216; McVail, p. 36; Woodward, p. 15) confirmed by all after-experience, conduct such as this, in various walks of professional life, seems little short of criminal, and has now reached such appalling magnitude as to urgently call for Government interference.

If vaccination is to be a reality, and not merely something which leads its subjects into a fools' paradise, the State must ensure, by an inspection through properly-qualified officials, that it is carried out in all ranks of society in a thoroughly efficient manner.

